# Real-world Characterization and Use of Insertable Cardiac Monitor Remote Programming

**DOI:** 10.19102/icrm.2022.13112

**Published:** 2022-11-15

**Authors:** Deepa Mahajan, Kate Frost, Keith Herrmann, Regina McGee-Taylor

**Affiliations:** ^1^Boston Scientific, St. Paul, MN, USA; ^2^Huntsville Hospital Heart Center, Huntsville, AL, USA

**Keywords:** Cryptogenic stroke, insertable cardiac monitor, remote monitoring, remote programming, syncope

## Abstract

Remote device programming may enable workflow efficiencies and reduce resource strains on clinics as well as patients. Although the remote patient management ecosystem has evolved, several challenges remain, and the role of remote device programming for an insertable cardiac monitor (ICM) has yet to be described in a real-world setting. The purpose of this study was to characterize the initial real-world use of remote programming of an ICM. The cohort included 8,238 patients with the LUX-Dx™ ICM (Boston Scientific, Marlborough, MA, USA) during the first year of commercial use, which is also the first year that remote programming was available for an ICM. A descriptive review of reprogramming events revealed that 24% of devices were reprogrammed and that 82% of all reprogramming events occurred remotely. Over 74% of first reprogramming events occurred within the first 30 days following device insertion, and nearly 80% of devices only had 1 reprogramming event. These early data support the hypothesis that remote programming of an ICM is a clinically useful tool that may improve the clinical experience of device programming optimization, especially within the first month following device insertion.

## Introduction

Remote monitoring and management of patient care enables a reduction of in-clinic visits, which carries benefits for both clinicians and patients. The recent coronavirus disease 2019 (COVID-19) pandemic brought about a swift change in clinical care, including an increase in the use of and recommendations for remote management and telemedicine in most patient populations. Professional societies such as the Heart Rhythm Society (HRS), European Heart Rhythm Association, Asia Pacific Heart Rhythm Society, Latin American Heart Rhythm Society, American College of Cardiology, and American Heart Association specifically addressed the use of telemedicine for the care of cardiac electrophysiology (EP) patients and stated that remote patient management (RPM) may be here to stay well beyond the pandemic.^1^ Although the challenges of RPM include the lack of equity in Internet access^2^ and the potential loss of health care payer reimbursement following the pandemic,^3^ the benefits appear to extend beyond these limitations, and health care providers are approaching RPM technologies as a growing field that needs to be developed, not overlooked.

For arrhythmia diagnostics and monitoring, RPM may provide a simple way to review episodes and finely tune device settings without requiring patients to come into the office. Insertable cardiac monitors (ICMs) provide long-term monitoring for patients with known or suspected cardiac arrhythmias. Recent models allow clinicians to deviate from default settings, which promotes individualized care and gives clinicians the ability to tailor monitoring for specific arrhythmia sensitivities, durations, etc. However, bringing patients into the clinic to adjust settings is a relatively minor task that confers an unnecessary burden to clinician schedules as well as a patient’s schedule and transportation needs. The ability to accurately and efficiently reprogram device settings remotely may reduce the burden on clinicians while improving both patient care and the patient experience.

The LUX-Dx™ ICM (Boston Scientific, Marlborough, MA, USA) enables remote programming of settings that correspond to the detection of several arrhythmias (atrial fibrillation [AF], pauses, tachycardias, bradycardias, and atrial tachycardias [AT]). Although the use and benefits of remote programming of cardiac implantable electronic devices (CIEDs) have been described, most notably in the context of the COVID-19 pandemic^4^ and future directions,^5^ the real-world use of this technology in an ICM has not been widely explored in the literature. The purpose of this investigation was to characterize the real-world use of remote programming for the identification and monitoring of arrhythmias during the first year of commercial use of the LUX-Dx™ ICM.

## Methods

### Study cohort

This study included all patients in the United States implanted with the LUX-Dx™ ICM between July 2020 and June 2021. Reasons for monitoring in this cohort included ventricular tachycardia, syncope, suspected AF, palpitation, other (seizures), oral anticoagulation change/AF management, cryptogenic stroke/AF, and AF pre–post ablation. Datasets from the LATITUDE Clarity™ database (Boston Scientific) were de-identified prior to the analysis and publication.

### The device

The LUX-Dx™ ICM is a small, leadless electronic device designed to monitor, record, and store data related to cardiac arrhythmias that fall into 5 categories: pauses, bradyarrhythmias, tachyarrhythmias, AF, and AT. The algorithm for each category contains settings that can be programmed as described in the product’s user manual. The LUX-Dx™ ICM is the first known commercially available ICM to provide remote programming capabilities. This investigation reviewed reprogramming events for algorithms in all categories.

### Characterization of use

The purpose of this investigation was to describe and characterize the use of remote programming of an ICM in a nationwide dataset. This includes the overall use of reprogramming, including the length of time until first reprogramming and the percentage of all devices that were reprogrammed as well as the frequency of reprogramming instances over time. Further analyses provide data describing the percentage of first reprogramming events that were completed remotely, the percentage of all reprogramming events that were made remotely, and the percentage of patients with devices that were remotely programmed.

### Identification of remote programming events

Reprogramming events that occurred remotely were identified with the LATITUDE Clarity™ database using timestamps corresponding to the time at which the programming change was confirmed on the device. Changes that were confirmed between 12:00 am and 6:00 am in the clinic’s local time zone were identified as a reliable surrogate of remote programming. All other events were considered to have occurred in the clinic. This differentiation was made using the rationale that events occurring in-clinic would transfer to the device during business hours or shortly afterward. Anything occurring between 12:00 am and 6:00 am is most likely remote because it is being transferred to the device during the initial daily communications and before clinic actions are likely to take place. This is a conservative measure of identifying events that are remotely programmed. It is possible that there are remote programming events that were confirmed on the device during the day (ie, between 6:00 am and 12:00 am) if the device was not in communication with the server overnight (ie, between 12:00 am and 6:00 am).

### Data governance and ethics

All data used for these analyses were pulled retrospectively from the LATITUDE Clarity™ database and de-identified, with approval from the company’s Data Governance Board and the permission of the data owners and in accordance with the U.S. Health Insurance Portability and Accountability Act and regulations thereunder. No information was obtained through intervention with patients for research purposes, so this analysis did not constitute human subject research as defined by the U.S. Department of Health and Human Services Office for Human Research Protections 45 CFR part 46.

## Results

**Figure 1:**
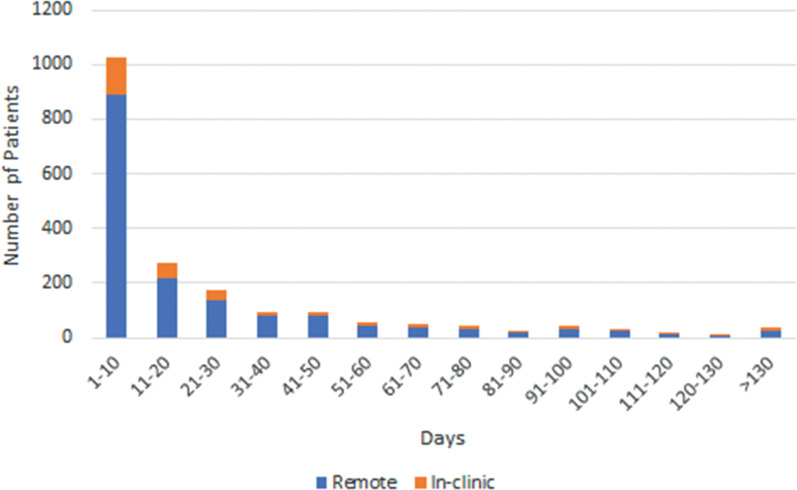
Time (days) from initial device programming at device insertion to the first reprogramming event for remote (blue) versus in-clinic (orange) programming.

Data from 8,238 patients with the LUX-Dx™ ICM were reviewed. These patients came from 803 clinics across the United States, and the average follow-up duration was 182 days. Of these patients, 1,974 (24%) had devices that were reprogrammed, and the average follow-up duration in this group was 217 days. **Figure 1** illustrates the length of time between initial programming (at device insertion) and the first reprogramming event for each patient. Over half of the patients (n = 1,025) experienced their first reprogramming event within the first 10 days following device insertion, of which 891 (87%) were remote and 134 (13%) were in-clinic, and 1,472 (74.5%) had their first reprogramming event within the first 30 days, of which 1,247 (85%) were remote. Only 39 (2.0%) patients had devices with a first reprogramming event that occurred >130 days following device insertion, of which 25 (64%) were remote. Across all first reprogramming events, 1,649 (83.5%) events were completed remotely.

**Figure 2:**
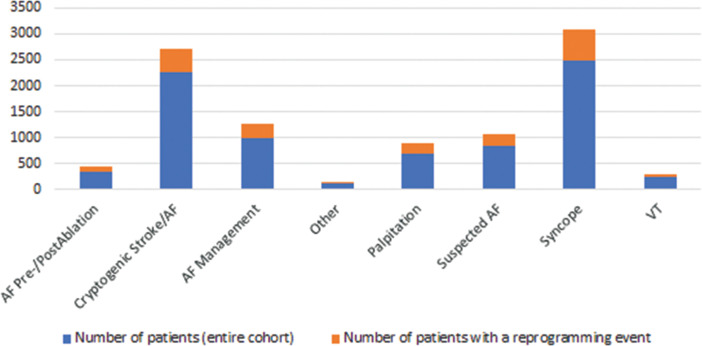
The distribution pattern of reason for monitoring for patients with devices that were reprogrammed (orange) is similar to the pattern that is seen across all patients (blue). *Abbreviations:* AF, atrial fibrillation; VT, ventricular tachycardia. “Other” includes seizures.

**Figure 2** provides the pattern of distribution of the reason for monitoring for each patient with a device that was reprogrammed as well as for the entire cohort. Most reprogramming events occurred in patients being monitored for syncope or cryptogenic stroke/AF. The pattern of distribution across reasons for monitoring is similar between the entire cohort and those who had a reprogramming event. Within each reason for monitoring, the percentage of patients with devices that were reprogrammed ranged from 19%–28%, which indicates that reprogramming events occurred evenly across all reasons for monitoring. The similar distribution patterns also indicate that the subgroup of patients with devices that were reprogrammed adequately represents the overall population of patients with the LUX-Dx™ ICM and that the reason for monitoring and associated default settings may not have an obvious impact on whether or not a device will be reprogrammed.

**Figure 3:**
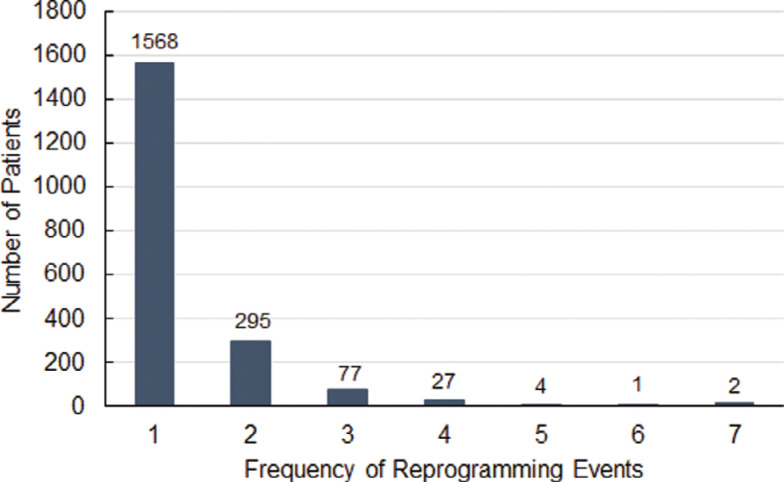
The frequency of reprogramming events per patient. Most patients experienced 1 device reprogramming event and only 7 individuals experienced >4 device reprogramming events.

A total of 4,902 reprogramming events occurred across the subgroup of 1,974 patients. Of these, 4,029 (82%) events were completed remotely. **Figure 3** illustrates the frequency of reprogramming events that occurred at the patient level. Most patients (n = 1,568, 79.4%) only underwent 1 reprogramming event, with an additional 295 patients experiencing only 2 reprogramming events. The maximum number of reprogramming events observed was 7, which occurred in 2 patients.

Individuals monitored for cryptogenic stroke and syncope included the majority of those who experienced a reprogramming event. Data from a subset of randomly chosen 20 individuals from each group were reviewed to assess for patterns in parameters being changed in-clinic and remotely. In the randomly selected 20 individuals monitored for cryptogenic stroke, the most frequent changes were a decrease in AF episode duration (9/10 experienced a decrease in AF duration to 2 min) and a decrease in the bradyarrhythmia rate (with a decrease from 40 bpm to 30 bpm in all 6 cases) **(Table 1)**. Forty of 48 (83.3%) reprogramming events were completed remotely, with a wide variety in the types of adjustments made remotely compared to in-clinic. However, all AT adjustments were made remotely. In the 20 randomly selected individuals monitored for syncope, the most common adjustment was a decrease in the bradyarrhythmia rate (6/7 decreased to 30 bpm) **(Table 2)**. Thirty of 37 (81.1%) reprogramming events were completed remotely. Again, there was a wide variety in types of adjustments made remotely, indicating an ability and clinical usefulness for remotely programming all parameters.

## Discussion

This investigation describes the initial real-world use of remote programming of an ICM using data from a large sample of patients with the LUX-Dx™ ICM. The LUX-Dx™ was the first commercially approved ICM in the United States to enable remote reprogramming; hence, these initial data provide an early look at utilization of a feature that clinicians have not had before. We found that nearly 25% of all patients had devices that were reprogrammed and that most reprogramming events occurred remotely. The relatively low percentage of devices undergoing reprogramming may support the usefulness of default settings or it may reflect the use rate of “early adopters” for a new technology that has not yet attained widespread use. Nonetheless, this initial characterization of ICM remote programming highlights a new interplay of technology, clinic workflow, and patient care.

Recent studies indicate that ICMs are plagued by high false-positive transmissions.^6,7^ One solution may be the ability to tailor device settings by allowing clinicians to reprogram these settings based on the number and types of episodes that they see and would like to see. However, the need to bring patients in-clinic for a reprogramming event and having a programmer available confer unintended consequences to a clinician’s decision by taking into account the potential inconvenience of an in-clinic visit as well as the possibility that a patient will not be able or willing to return to the clinic when reprogramming would have the greatest benefit. As observed, when remotely programming CIEDs for magnetic resonance imaging scans, remote communication with cardiac devices is effective and contributes to patient satisfaction.^8^ A care pathway and device that allow for remote reprogramming may reduce the clinic burden and ensure that device settings are optimized in a timely manner. This aspect not only improves clinical efficiency but may improve patient care as well.

The intent of remote programming is to improve clinical efficiency and patient care without imposing an additional workload. This study found that >50% of the reprogramming events occurred within the first 10 days post-insertion, indicating a need for only a short window of time to identify and implement optimal settings. In conjunction with the findings that 79.4% of patients only experienced 1 reprogramming event and that 83.5% of first reprogramming events occurred remotely, it appears that the most common usage may be a single remote reprogramming event within the first 10 days following device insertion.

The types of programming changes observed in this study appear logical to the intent of ICM use in these patient cohorts. Clinic-specific or “out-of-the-box” settings cannot take into account the myriad of possibilities for programming selection; for example, a tachycardia detection of 150 bpm in a young, healthy man may result in multiple detections that may not be actionable, while that same detection in an 80-year-old man may have clinical significance. Clinicians are often looking for any duration of AF in cryptogenic stroke patients; thus, the duration of the event may need to be shortened. Bradyarrhythmia and pause detections may need to be adjusted on a patient-by-patient basis, which includes the observed general increase in bradyarrhythmia duration that may represent a need to reduce clinically non-actionable episodes. The ability to remotely program an ICM allows the clinician to quickly and easily individualize detections and improve the quality of data that are transmitted.

The use and importance of telemedicine and remote care were significantly enhanced during the COVID-19 pandemic. Although this likely affected the prevalence of remote monitoring observed in this study, it is expected to remain relevant once the pandemic subsides. Prior to the pandemic, technologies supporting RPM were on the rise. In 2011, Movsowitz and Mittal discussed the introduction of remote data transmission in implantable devices, imploring clinicians to begin adjusting workflows to accommodate the shift in patient care and follow-up being driven by RPM.^9^ In 2015, the Heart Rhythm Society released an expert consensus concluding that remote monitoring will be the new standard of care for patients with CIEDs.^10^ In 2019, Atreja et al. reviewed the evolution of RPM specific to EP, noting that frequent RPM increases the quality of life and positive outcomes for certain populations.^11^ Considering the interest and technological advances that occurred before the pandemic, it is clear that the pandemic may have acted as a springboard and that continued improvements to the RPM ecosystem, such as remote device programming, are warranted and require further studies to establish evidence-based guidelines.

### Limitations

This study is limited by the use of retrospective device data that do not provide clinical decision-making, rationale, or the clinician experience. Furthermore, these data do not provide patient-level demographics or comorbidities that may further explain why some reprogramming events occur remotely or in-clinic. It is to be noted that a proportion of remote programming events identified in this study are likely lower than remote programming happening in the real world as a conservative surrogate is used to identify a reprogramming event as remote. It is possible that some reprogramming events were confirmed on the device between 6:00 am and 12:00 am and thus misclassified in this current analysis. We expect that this is a low number. Moreover, reprogramming data from a random subset of 20 individuals from each group were reviewed, which is a small sample size. This study is also limited by the use of data collected during the time period of the COVID-19 pandemic, which likely facilitated an increase in the use of remote programming. However, the use of remote programming is expected to remain at this level or increase beyond the pandemic period.

## Conclusion

This study characterized the initial use of remote programming in a cohort of 8,238 patients with the LUX-Dx™ ICM. Reprogramming occurred in 24% of this cohort, and 82% of all reprogramming events occurred remotely. Nearly 80% of patients only experienced 1 reprogramming event, and most reprogramming events took place within the first 30 days following device insertion. These data support the hypothesis that remote programming of an ICM is a clinically useful tool that may improve the clinical experience of device programming optimization, especially within the first month following device insertion.

## Figures and Tables

**Table 1:** Parameters and Methods of Adjustment for Cryptogenic Stroke

	Parameter Adjustments			Method	
	Increase	Decrease	Total*	Remote	In-clinic
AF					
Episode duration (n = 12)	2	10	12	10	2
Sensitivity (n = 4)	3	1	4	4	0
AT					
Duration (n = 3)	1	2	3	3	0
Rate (n = 1)	1	0	1	1	0
Brady					
Duration (n = 4)	4	0	4	4	0
Rate (n = 7)	1	6	7	6	1
Sensing					
Sensitivity floor (n = 3)	2	1	3	3	0
Total refractory (n = 1)	1	0	1	0	1
Pause					
Duration (n = 2)	2	0	2	1	1
Sensitivity (n = 3)	3	0	3	2	1
VT					
Rate (n = 5)	1	4	5	4	1
Sensitivity (n = 3)	1	2	3	2	1

*Abbreviations:* AF, atrial fibrillation; AT, atrial tachycardia; VT, ventricular tachycardia. The number of patients with devices that were reprogrammed is represented by n = X. *Number represents the total number of adjustments.

**Table 2:** Parameters and Methods of Adjustment for Syncope

	Parameter Adjustments			Method	
	Increase	Decrease	Total*	Remote	In-clinic
AF					
AF enable (n = 2)	0	2	2	1	1
Episode duration (n = 1)	0	1	1	1	0
AT					
AT enable (n = 1)	0	1	1	1	0
Duration (n = 1)	1	0	1	1	0
Brady					
Duration (n = 4)	4	1	5	4	1
Rate (n = 7)	2	7	9	8	1
Sensing					
Sensitivity floor (n = 6)	1	6	7	5	2
Total refractory (n = 1)	2	0	2	2	0
Pause					
Duration (n = 2)	1	1	2	2	0
VT					
Rate (n = 5)	2	3	5	3	2
Sensitivity (n = 1)	0	1	1	1	0

*Abbreviations:* AF, atrial fibrillation; AT, atrial tachycardia; VT, ventricular tachycardia. The number of patients with devices that were reprogrammed is represented by n = X. *Number represents the total number of adjustments.
